# Global Analysis of the Co‐Occurrence of Antimicrobial and Metal Resistance Genes in Bacterial Genomes From Dairy‐Isolated Pathogens

**DOI:** 10.1111/1462-2920.70285

**Published:** 2026-04-01

**Authors:** Arlen Carvalho de Oliveira Almeida, Hannay Crystynah Almeida de Souza, Juliana Fidelis, Anamaria Mota Pereira dos Santos, Ana Beatriz Portes, Uiara Moreira Paim, Paloma Almeida Rodrigues, Marion Pereira da Costa, Carlos Adam Conte‐Junior

**Affiliations:** ^1^ Center for Food Analysis (NAL), Technological Development Support Laboratory (LADETEC) Federal University of Rio de Janeiro (UFRJ), Cidade Universitária Rio de Janeiro Rio de Janeiro Brazil; ^2^ Laboratory of Advanced Analysis in Biochemistry and Molecular Biology (LAABBM), Department of Biochemistry Federal University of Rio de Janeiro (UFRJ), Cidade Universitária Rio de Janeiro Rio de Janeiro Brazil; ^3^ Analytical and Molecular Laboratorial Center (CLAn) Institute of Chemistry (IQ), Federal University of Rio de Janeiro (UFRJ), Cidade Universitária Rio de Janeiro Rio de Janeiro Brazil; ^4^ Graduate Program in Food Science (PPGCAL), Institute of Chemistry (IQ), Federal University of Rio de Janeiro (UFRJ), Cidade Universitária Rio de Janeiro Rio de Janeiro Brazil; ^5^ Laboratory of Technology and Inspection of Milk and Derivatives (LaITLácteos) School of Veterinary Medicine, Federal University of Bahia (UFBA), Ondina Salvador Bahia Brazil; ^6^ Graduate Program in Biochemistry (PPGBq), Institute of Chemistry (IQ), Federal University of Rio de Janeiro (UFRJ), Cidade Universitária Rio de Janeiro Rio de Janeiroa Brazil; ^7^ Graduate Program in Veterinary Hygiene (PGHigVet), Faculty of Veterinary Medicine, Fluminense Federal University (UFF) Niterói Rio de Janeiroa Brazil; ^8^ Laboratory of Microorganism Structure, Department of General Microbiology Institute of Microbiology Paulo de Góes (IMPG), Federal University of Rio de Janeiro Rio de Janeiro Brazil; ^9^ Graduate Program in Chemistry (PGQu), Institute of Chemistry (IQ), Federal University of Rio de Janeiro (UFRJ), Cidade Universitária Rio de Janeiro Rio de Janeiroa Brazil

**Keywords:** contaminant‐driven resistance, co‐selection, foodborne bacterial adaptation, milk safety assessment, one health

## Abstract

Metal contamination in dairy production can drive antimicrobial resistance co‐selection independently of antibiotic use, yet the genomic architecture linking metal resistance and antimicrobial resistance across dairy‐isolated pathogens remains largely uncharacterized globally. We analysed 3303 genomes from dairy environments across 52 countries, creating the first genome‐resolved map of co‐occurring resistance determinants in this critical agricultural interface. Metal/stress resistance genes were present at levels comparable to AMR genes (28.85% of isolates showed metal dominance), with arsenic (*arsB*: 42.6% prevalence) and copper/silver systems (*silR*: 98% in *Klebsiella pneumoniae* ) frequently co‐occurring with tetracycline (*tet*(38): 43%) and multidrug efflux determinants (*lmrS*: 43%). Statistical analysis of 29,299 gene pairs identified 523 significant associations (FDR < 0.05), revealing genetic linkage between arsenic resistance and tetracycline/multidrug efflux genes (Jaccard ≥ 0.98) in over 40% of isolates. Co‐occurrence burden varied ninefold across taxa, with *K. pneumoniae* functioning as a super‐reservoir species. Functional network analysis revealed that arsenic and copper resistance families operate as genomic hubs connecting to multiple antimicrobial classes, demonstrating that metal exposure maintains entire resistance modules through genetic linkage. These findings establish that AMR control strategies focused solely on antibiotic stewardship are insufficient; effective resistance mitigation requires integrated management of environmental metal contamination in dairy production systems.

## Graphical abstract

Metal contamination in dairy systems drives antibiotic resistance through co‐selection. Analysis of 3303 genomes revealed 74% of isolates harbour more metal than AMR genes. Arsenic (*arsB*) and tetracycline (*te*t(38)) resistance co‐occur in 43% of bacteria, exhibiting nearly identical prevalence, suggesting a tight genetic linkage.

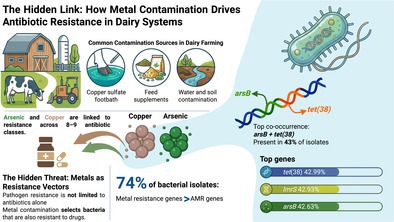

## 1 Introduction

Genes conferring resistance to metals and other environmental stressors often outnumber antimicrobial resistance genes in bacterial genomes (de Souza et al. 2025). Recognizing this can help public health professionals appreciate the long‐term impact of environmental trace metals, making them feel the importance of addressing these persistent pressures. Despite this, current surveillance and mitigation strategies remain primarily focused on antibiotic use, potentially overlooking critical drivers of bacterial persistence and co‐selection (Wales and Davies 2015; Vats et al. 2022; Collis et al. 2024). This imbalance overlooks a fundamental feature of dairy production systems: the selective pressures imposed by trace metals, which persist in the environment far longer than antimicrobial residues and continue to influence bacterial populations even in the absence of direct therapeutic interventions (Qi et al. 2021).

Metals introduced through feed, footbaths, and environmental contamination exert a continuous selection pressure on bacterial populations, one that biodegradable antibiotics cannot exercise (Tian et al. 2025). These selective pressures coincide with the widespread detection of toxic elements, such as arsenic, lead, and chromium, in raw milk, which has been documented across multiple regions worldwide (Almeida et al. 2025). In some cases, including recent assessments of buffalo milk, reported concentrations have surpassed regulatory limits and indicate non‐negligible health risks, underscoring the ecological and public health relevance of metal exposure within dairy production systems, emphasizing the need for policy adjustments to address environmental metal pollution as a critical component of public health strategies (Shahzad et al. 2025).

At the molecular level, interactions between metals and antimicrobials often arise through interconnected resistance mechanisms. Broad‐substrate efflux pumps exemplify cross‐resistance, simultaneously expelling both antimicrobials and metals, thereby establishing a shared pathway for bacterial survival under multiple selective pressures (Gillieatt and Coleman 2024; Gaurav et al. 2023). Mobile genetic elements often carry metal and antibiotic resistance genes in physical linkage, promoting co‐resistance. In addition, metal exposure activates stress‐response pathways that co‐regulate antimicrobial resistance mechanisms (Xu and Lin 2024; Murray et al. 2024). Recent transcriptomic work demonstrates that copper can induce efflux and detoxification systems associated with reduced susceptibility to multiple antibiotic classes, supporting the idea that metal exposure drives broader resistance phenotypes (Marcos‐Torres et al. 2025). Environmental studies reinforce this concept by showing that in mining‐impacted landscapes, metal contamination alters microbial community structure and elevates health‐risk profiles associated with antimicrobial resistance, even when the total abundance of antibiotic resistance genes (ARGs) is comparable to that in uncontaminated soils (Hou et al. 2025). Likewise, metagenomic analyses of antibiotic‐exposed activated‐sludge systems show that antimicrobial pressure increases gene mobility and promotes co‐selection with metal‐detoxification pathways, with specific antibiotics exerting notably strong co‐selective effects (Zhao et al. 2025). Historical genomic data further indicate that the co‐localisation of lactose metabolism genes, metal‐detoxification operons, and colistin‐resistance genes on plasmids has been present in dairy facilities since at least the 1980s, suggesting that metal‐driven co‐selection has shaped dairy‐associated genomes for decades (Kröger et al. 2023; Portes et al. 2022).

The bacterial taxa most frequently isolated as pathogens within dairy herds, particularly *Staphylococcus aureus* , *Escherichia coli* , and *Klebsiella pneumoniae* , illustrate how these pressures unfold across ecological contexts (Ágredo‐Campos et al. 2023). These organisms occupy distinct niches along the farm‐to‐fork continuum and experience heterogeneous exposure to antimicrobials, metals, and chemical stressors. Genomic reconstructions suggest that bovine‐associated *S. aureus* emerged through multiple independent host‐jump events from humans, followed by clonal expansion that coincided with the intensification and globalisation of dairy farming (Yebra et al. 2022). Recent genomic surveys of *E. coli* and other *Enterobacteriaceae* from dairy systems reveal pronounced intra‐host diversity and complex multidrug‐resistance profiles, which vary significantly across regions and influence local resistance spread (Alencar et al. 2025; Moradzadeh et al. 2025). Comparative genomic analyses across different geographic areas also demonstrate substantial variation in accessory‐gene sharing, horizontal gene‐transfer rates, and mobilome composition, shaping the regional ecology and resistance dissemination patterns (Mouftah et al. 2022). Despite advances in resistome characterisation, no study has evaluated the genomic architecture of metal/stress and antimicrobial resistance determinants across a globally representative panel of dairy‐associated isolates. Existing surveillance efforts have either focused on single pathogens, narrow geographic regions, or isolated mechanisms such as disinfectant tolerance, leaving metal–antimicrobial co‐resistance largely unaddressed (Ivanova et al. 2025; Yebra et al. 2022; Mouftah et al. 2022). Addressing this gap can inspire the community to recognise their role in advancing comprehensive resistome knowledge, as, the central question of whether metal and stress‐resistance systems co‐occur with antimicrobial resistance determinants at rates exceeding random expectation, and whether these patterns vary predictably among bacterial lineages, remains unresolved.

To address this gap, we analysed 3303 bacterial genomes from dairy environments spanning 52 countries and nearly three decades (1990s–2020s). By integrating curated antimicrobial and metal/stress resistance annotations with extensive metadata on geography, source, and host, we mapped the global burden of resistance determinants in major dairy‐isolated pathogen taxa, quantified the statistical structure of gene co‐occurrence, and assessed how these associations are functionally organised within antimicrobial classes and metal‐resistance families. This genome‐resolved framework provides a foundation for understanding how environmental metal exposure and antimicrobial use jointly shape resistance in dairy systems and identifies resistance modules. Furthermore, this study highlights the environmental impacts on resistance development, encouraging policymakers and microbiologists to consider ecological factors in resistance management and intervention planning.

## 2 Methods

### 2.1 Genome Retrieval and Dataset Construction

Bacterial genomes of pathogens isolated from the dairy production chain were retrieved from the NCBI Pathogen Detection Isolates Browser (https://www.ncbi.nlm.nih.gov/pathogens/isolates) between July and September 2025. A multi‐step filtering process was applied to construct the final dataset. Initial screening retained isolates with explicit association to milk, dairy products, dairy animals, or dairy‐processing environments. Subsequently, genomes were restricted to 10 bacterial genera of relevance to dairy production and safety (*Staphylococcus, Escherichia, Klebsiella, Salmonella, Listeria, Streptococcus, Campylobacter, Bacillus, Enterococcus* and *Pseudomonas*). To ensure robust downstream structural analysis and prevent misinterpretation due to poor assembly quality, genome selection employed strict contiguity thresholds: genomes with more than 500 contigs and N50 values below 10 kb were systematically excluded, in accordance with NCBI's standard parameters. The resulting refined dataset demonstrated high sequence contiguity, featuring an overall median N50 of 176,966 bp and a median of 56 total contigs per genome.

Finally, isolates lacking complete metadata (species‐level identification, geographic origin, year of isolation, or sample source) were excluded. No explicit de‐replication or genomic redundancy filtering (e.g., removal of outbreak‐related clones) was performed. All retrieved genomes meeting the metadata inclusion criteria were preserved to capture both horizontal gene transfer–mediated associations and lineage‐driven amplification of naturally occurring resistance modules in the dairy ecosystem. This sequential filtering yielded a final curated dataset of 3303 dairy‐isolated pathogen genomes distributed across 52 countries and representing 10 bacterial species. Metadata were harmonised using standardised classifications for countries, host organisms and dairy sample categories.

### 2.2 Annotation of Antimicrobial and Metal/Stress‐Resistance Determinants

Annotations provided by the NCBI Pathogen Detection pipeline, which utilises natively AMRFinderPlus (using predominantly version 4.0.23, varying dynamically per NCBI's periodic pipeline releases across the analytical timeframe) to identify antimicrobial resistance genes (ARGs) and stress‐associated determinants (MRGs), were employed in our analyses. Identification relied upon standard NCBI algorithmic thresholds, maintaining an optimised combination of HMM profile scores, a minimum of > 90% sequence coverage, and > 90% sequence identity to the query databases. Within the context of our investigation, MRGs/SRGs were strictly defined according to the AMRFinderPlus functional hierarchy to explicitly encompass metal resistance, biocide tolerance, and specific physiological stress responses (e.g., acid and heat stress). All analyses relied exclusively on these standardised annotations. For each genome, resistance profiles were expressed as binary presence–absence matrices, allowing for the quantification of gene prevalence and the construction of genome‐level resistance burdens. No additional annotation pipelines or external databases were applied.

### 2.3 Quantification of Resistance Burdens

For each genome, the total number of ARGs and MRGs was calculated by summing the respective binary indicators. Gene‐level prevalence was determined as the proportion of genomes carrying each determinant, allowing for the identification of the most frequent ARGs and MRGs/SRGs circulating in dairy‐isolated pathogens. These data supported all descriptive comparisons among bacterial species, sample sources, and geographic regions.

### 2.4 Gene Co‐Occurrence Analysis

To investigate whether antimicrobial and metal/stress resistance determinants tend to co‐occur within individual bacterial genomes, pairwise co‐occurrence was assessed for every combination of ARGs and MRGs/SRGs across the 3303 isolates. For each gene pair, co‐occurrence metrics were derived directly from presence–absence patterns. Statistical significance was evaluated using Fisher's exact test, chosen for its suitability for sparse and binary genomic data. *p*‐values were corrected for multiple comparisons using the Benjamini–Hochberg false discovery rate (FDR) procedure. Associations were considered significant when the FDR‐adjusted *p*‐value was below 0.05, and the direction of the odds ratio indicated a positive association. The magnitude of co‐occurrence was additionally summarised using the Jaccard similarity coefficient derived from the same presence–absence patterns. For each genome, the co‐occurrence count index was calculated to quantify the normalised empirical odds of simultaneous MRG/AMR detection per genome, ensuring it operates as a standardised cross‐comparative analytical tool regardless of the intrinsic species being studied (represented numerically as the number of significant ARG–MRG/SRG associations present within that isolate).

### 2.5 Functional Aggregation of Resistance Determinants

To examine patterns of co‐occurrence at broader biological scales, individual genes were grouped into functional categories representing antimicrobial classes and metal/stress‐resistance families. Importantly, all respective gene‐to‐class assignments stringently adhered to their originating AMRFinderPlus ontology classifications; no external manual curation or subjective consolidation was imposed that might alter the hierarchical definitions set by the NCBI pipeline, thereby preserving maximum methodological reproducibility. A genome was considered positive for a given class or family/mechanism when at least one corresponding determinant was detected. Co‐occurrence between antimicrobial classes and metal‐resistance families was quantified using the same statistical framework described above. This approach enabled the identification of preferential class–family pairings and broader co‐resistance signatures characteristic of dairy‐associated bacterial populations.

### 2.6 Statistical Framework

All statistical analyses were performed using R (version 4.4.1). Descriptive statistics summarised genomic, geographic, and resistance‐profile distributions. Fisher's exact test was used for all inferential analyses of co‐occurrence, and multiple testing was controlled using the Benjamini–Hochberg FDR method. Odds ratios and Jaccard coefficients were computed from the same contingency structures.

## 3 Results

### 3.1 Sample Sources, Global Distribution and Temporal Data

**FIGURE 1:**
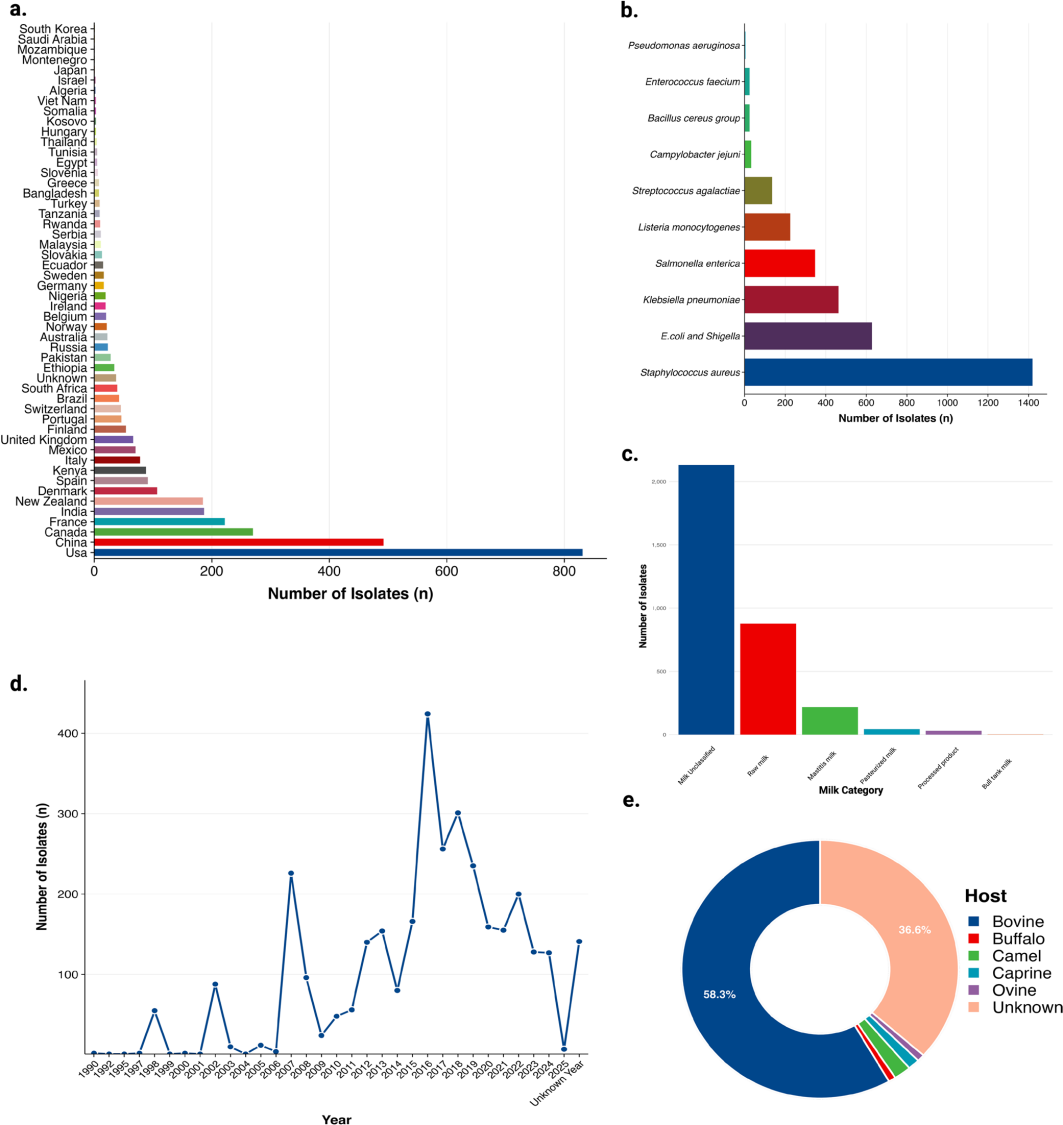
Characterisation of bacterial isolates from milk and dairy products worldwide. (a) Geographic distribution of 3303 bacterial isolates across 52 countries. Horizontal bars represent the number of isolates per country, ranked in descending order. The top five countries (USA, China, Canada, France and India) collectively contributed 60.61% of all isolates; (b) Distribution of isolates across 10 bacterial groups. Bars indicate the absolute frequency of each taxonomic group, with *Staphylococcus aureus* (42.99%), *E. coli* and *Shigella* (19.01%) and *Klebsiella pneumoniae* (14.02%) being the most prevalent; (c) Classification of isolates by milk source category. Six categories are shown: Milk Unclassified (64.52%), Raw milk (26.55%), Mastitis milk (6.60%), Pasteurised milk (1.30%), Processed product (0.91%) and Bulk tank milk (0.12%); (d) Temporal distribution of isolate collection from 1990 to 2025. The time series plot displays the number of isolates collected per year, with peaks in 2016 (12.84%), 2018 (9.11%) and 2017 (7.75%). Isolates with unknown collection year (*n* = 141) were excluded; (e) Distribution of isolates by host species. The pie chart shows the proportion of isolates derived from bovine (58.28%), unknown hosts (36.60%), camel (2.00%), caprine (1.39%), ovine (0.88%) and buffalo (0.85%).

The final dataset comprised 3303 bacterial genomes isolated from milk and dairy products (Table S1). Isolates were distributed across six sample source categories (Figure 1C, Table S3), although representation was unbalanced. The majority were classified as ‘Milk Unclassified’ (*n* = 2131, 64.52%), a category encompassing isolates lacking granular source metadata in the original submissions. Raw milk represented the second‐largest source (*n* = 877, 26.55%), followed by milk from mastitis (*n* = 218, 6.60%). More processed or controlled sources, pasteurised milk (*n* = 43, 1.30%), processed dairy products (*n* = 30, 0.91%), and bulk tank milk (*n* = 4, 0.12%), collectively accounted for less than 3% of isolates, reflecting the limited availability of genomes from these sampling contexts.

The metadata of hosts indicated a predominance of bovine‐associated isolates (*n =* 1925, 58.28%), consistent with dairy cattle as the primary production animal (Figure 1E, Table S6). Understanding this distribution is necessary for insights into pathogen ecology and host specificity. However, over one‐third of isolates (*n =* 1209, 36.60%) lacked host identification, which hampers full interpretation of host‐pathogen relationships. The remaining isolates were derived from camelid (*n =* 66, 2.00%), caprine (*n =* 46, 1.39%), ovine (*n =* 29, 0.88%), and buffalo (*n =* 28, 0.85%) sources.

The geographic distribution of isolates encompassed 52 countries (Figure 1A, Table S1), although representation was uneven. The United States contributed the largest share (*n =* 831, 25.16%), followed by China (*n =* 492, 14.90%), Canada (*n =* 270, 8.17%), France (*n =* 222, 6.72%), and India (*n =* 187, 5.66%). Five additional countries contributed more than 75 isolates each: New Zealand (*n =* 185, 5.60%), Denmark (*n =* 107, 3.24%), Spain (*n =* 91, 2.76%), Kenya (*n =* 88, 2.66%), and Italy (*n =* 78, 2.36%). The remaining 42 countries each contributed fewer than 75 isolates, with 34 countries providing fewer than 20 genomes. A small fraction of isolates (*n =* 37, 1.12%) lacked geographic attribution and were classified as ‘Unknown Country.’ This geographic distribution largely reflects surveillance capacity and genome sequencing resources rather than proportional representation of global dairy production. The United States and China, which collectively account for approximately 25% of global milk production, contributed 40% of genomes in this dataset. Conversely, major dairy‐producing regions in South America and Africa were underrepresented, reflecting disparities in genomic surveillance infrastructure rather than actual variation in dairy‐isolated pathogens populations.

Temporal distribution demonstrated marked variation (Figure 1D, Table S4), with genome submissions peaking in 2016 (*n =* 424, 12.84% of the dataset). Other high‐contribution years included 2018 (*n =* 301, 9.11%), 2017 (*n =* 256, 7.75%), 2019 (*n =* 235, 7.11%), and 2007 (*n =* 226, 6.84%). A total of 141 isolates (4.27%) lacked temporal metadata.

### 3.2 Taxonomic Composition

Taxonomic analysis revealed concentration within 10 bacterial species (Figure 1B, Table S2). *S. aureus* predominated, accounting for nearly half of all isolates (*n =* 1420, 42.99%). Three additional taxa comprised most of the remaining dataset: *E. coli* and *Shigella* (*n =* 628, 19.01%), *Klebsiella pneumoniae* ( *K. pneumoniae* ) (*n =* 463, 14.02%) and *Salmonella enterica* ( *S. enterica* ) (*n =* 347, 10.51%). These four groups collectively represented 86.53% of all genomes.

The remaining six groups occurred at lower frequencies: *Listeria monocytogenes* ( *L. monocytogenes* ) (*n =* 225, 6.81%), *Streptococcus agalactiae* ( *S. agalactiae* ) (*n =* 136, 4.12*%), Campylobacter jejuni* ( *C. jejuni* ) (*n =* 32, 0.97%), *Bacillus cereus* (*n =* 24, 0.73%), *Enterococcus faecium* (*n =* 24, 0.73%) and *Pseudomonas aeruginosa* (*n =* 4, 0.12%). This taxonomic structure reflects a dataset dominated by major dairy pathogens, with a smaller representation of environmental and processing‐associated species.

### 3.3 Gene‐Level Landscape of Antimicrobial and Metal/Stress Resistance

**FIGURE 2:**
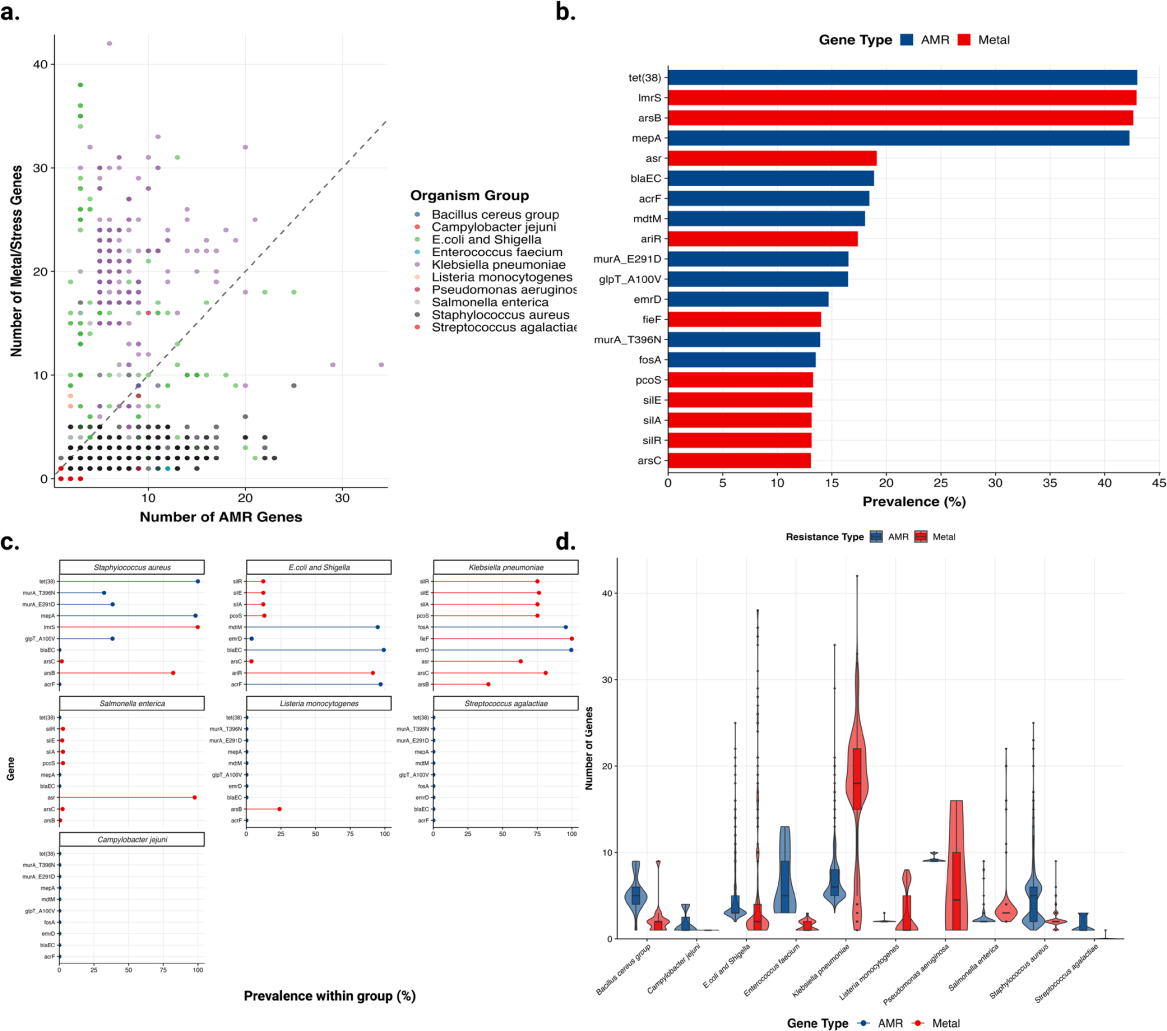
Characterisation of resistance gene burden and prevalence across bacterial isolates. (a) Relationship between antimicrobial resistance (AMR) and metal/stress resistance gene counts per isolate (*n* = 3303). Each point represents an individual isolate, coloured by bacterial group. The diagonal reference line (y = x) indicates an equal burden of both gene types. Mean AMR genes per isolate: 4.61 ± 3.42; mean metal/stress genes: 4.88 ± 6.84; (b) Prevalence of the 20 most common resistance genes across all isolates. Horizontal bars represent the percentage of isolates carrying each gene, with genes colour‐coded as AMR determinants (10 genes) or metal/stress resistance determinants (10 genes, marked in red). The most prevalent gene, tet(38), was present in 42.99% of isolates; (c) Top 10 resistance genes by bacterial group. Faceted lollipop plots show gene prevalence (%) within each bacterial group containing at least 30 isolates. Each panel represents a distinct bacterial group, with genes ranked by within‐group prevalence from highest (top) to lowest (bottom); (d) Distribution of resistance gene counts across bacterial groups. Combined violin and boxplots display the distribution of AMR genes (left) and metal/stress genes (right) for each bacterial group. Violins show the density distribution, while boxplots indicate median, quartiles, and outliers.

Resistance gene repertoires varied extensively across isolates (Figure 2A–D, Tables S1–S4). AMR gene counts ranged from 1 to 34 per isolate (mean = 4.61, SD = 3.42), while metal/stress gene counts spanned 0–42 (mean = 4.88, SD = 6.84). Notably, metal/stress genes outnumbered AMR genes in 953 isolates (28.85% of the dataset), creating a detectable asymmetry in the scatter plot distribution (Figure 2A). All isolates carried at least one AMR gene, whereas 135 isolates (4.09%) lacked metal/stress genes entirely (Table S1).

Prevalence analysis identified the most widespread resistance determinants (Figure 2B, Table S2). The top 20 genes included 10 AMR and 10 metal/stress determinants, with prevalence values ranging from 42.99% to 11.81%. The tetracycline efflux gene *tet*(*38*) ranked first (*n =* 1420, 42.99%), followed by the metal resistance transporter *lmrS* (*n =* 1418, 42.93%) and the arsenic efflux permease *arsB* (*n =* 1408, 42.63%). The multidrug transporter *mepA* occupied fourth position (*n =* 1396, 42.26%). Additional highly prevalent genes included the arsenate reductase *asr*‐tet(38) (*n =* 631, 19.10%), the beta‐lactamase *bla*_EC_ (*n =* 623, 18.86%), and the multidrug efflux systems *acrF* (*n =* 609, 18.44%) and *mdtM* (*n =* 596, 18.04%). The remaining top 20 genes showed prevalence between 17.38% and 11.81%, encompassing both AMR determinants (*murA*_E291D, *fosA*, *glpT*_A100V and *acrR*_M109I) and metal/stress genes (*ariR, fieF, crcB, ncrB, arsA, arsC* and *arsR*).

Gene prevalence patterns exhibited marked taxonomic variation (Figure 2C, Table S3). Among the seven groups with sufficient sample sizes (*n* ≥ 30, ensuring robust prevalence estimates), three distinct profiles emerged. *S. aureus* displayed near‐universal carriage of tetracycline and multidrug resistance genes: *tet(38)* (100.00%), *lmrS* (99.93%) and *mepA* (99.58%). In contrast, *K. pneumoniae* exhibited exceptionally high prevalence of copper/silver resistance operon components: *silR* (98.27%), *silE* (98.06%) and *silA* (97.84%). *L. monocytogenes* exhibited a distinct pattern, characterised by arsenic resistance, with the following genes being predominant: *arsB* (98.22%), *arsC* (96.00%) and *asr* (96.44%). *S. enterica* carried arsenic determinants at intermediate‐to‐high frequencies: *asr* (78.10%) and *arsB* (72.62%), alongside the efflux pump *acrF* (46.11%). *Streptococcus agalactiae* matched *S. aureus* in terms of tetracycline and multidrug resistance, with *tet*(38), *mepA*, and *lmrS* all at 100.00%. *E. coli* and *Shigella* demonstrated moderate prevalence of copper/silver systems: *silR* (31.85%), *silE* (31.69%) and *pcoS* (30.41%). *C. jejuni* displayed minimal gene carriage across all categories.

Quantitative gene burden analysis reinforced these taxonomic distinctions (Figure 2D, Table S4). Mean AMR gene counts varied 6.3‐fold across groups, ranging from 1.67 genes in *L. monocytogenes* (SD = 1.34) to 10.52 genes in *K. pneumoniae* (SD = 3.66). Intermediate values characterised *E. coli* and *Shigella* (7.39 genes, SD = 5.34), *S. aureus* (4.57 genes, SD = 2.22), *S. agalactiae* (4.13 genes, SD = 2.13) and *S. enterica* (2.55 genes, SD = 2.03). Metal/stress gene burdens showed even greater variation, spanning 12.1‐fold from 1.52 genes in *C. jejuni* to 20.22 genes in *L. monocytogenes* (mean = 20.22, SD = 6.43). *K. pneumoniae* carried the second‐highest metal burden (15.22 genes, SD = 6.21), followed by *E. coli* and *Shigella* (7.63 genes, SD = 7.29), *S. enterica* (5.78 genes, SD = 4.56), *S. aureus* (5.39 genes, SD = 2.08) and *S. agalactiae* (3.06 genes, SD = 1.52). These distributions establish substantial quantitative and qualitative differences in resistance architecture across major dairy‐isolated pathogen lineages.

Notably, genes exhibiting 100% intra‐species prevalence (e.g., specific intrinsic variants within particular lineages) indicate intrinsic, structural resistance rather than horizontally acquired resistance. Their ubiquitous presence fundamentally differs from that of genes under active environmental co‐selection pressure, and thus, they respond differently to environmental selective pressures than actively mobilized structural elements.

### 3.4 Co‐Occurrence Architecture Between AMR and Metal/Stress Resistance Genes

**FIGURE 3:**
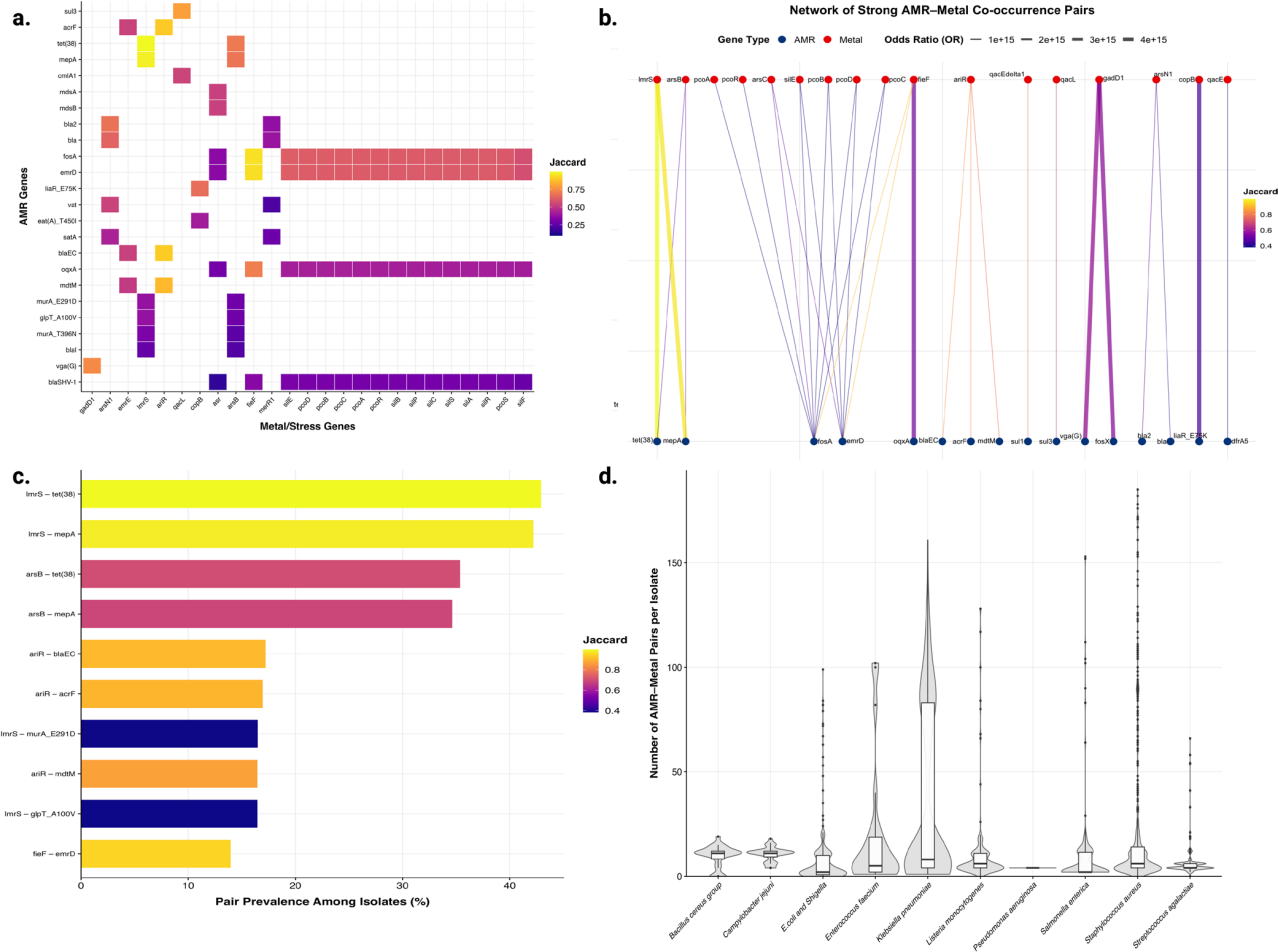
Co‐occurrence patterns between antimicrobial resistance (AMR) and metal/stress resistance genes across dairy‐isolated pathogens. (a) Heatmap of Jaccard similarity indices for significant AMR–metal gene pairs. Only pairs meeting the following criteria are shown: ≥ 5 isolates carrying both genes, Jaccard index ≥ 0.10, and FDR < 0.05 (Fisher's exact test with multiple‐testing correction). Rows represent AMR genes and columns represent metal/stress resistance genes. Colour intensity reflects the strength of co‐occurrence based on the Jaccard similarity index. (b) Bipartite network representation of a subset of the highest‐ranked significant AMR–metal gene pairs (selected based on Jaccard similarity to improve visualisation clarity). AMR genes are shown at the bottom (blue nodes) and metal/stress resistance genes at the top (orange nodes). Edge colour represents the Jaccard similarity index (co‐occurrence strength), whereas edge width reflects the odds ratio (OR) of co‐presence derived from Fisher's exact test, indicating the magnitude of statistical association between gene pairs. (c) Top 20 most prevalent AMR–metal gene pairs across all isolates. Horizontal bars represent the percentage of isolates simultaneously carrying both genes in each pair. Colour intensity indicates the corresponding Jaccard similarity index. The most frequent pair (*tet(38)–lmrS*) occurred in 42.9% of all genomes analysed. (d) Distribution of the number of significant AMR–metal co‐occurrence pairs per isolate, stratified by bacterial taxonomic group. Boxplots represent the per‐genome burden of significant co‐occurring pairs identified in the dataset. The central line indicates the median, boxes represent interquartile ranges, and whiskers extend to 1.5× IQR.

Pairwise analysis revealed extensive co‐occurrence between AMR and metal/stress genes (Figure 3A–D, Tables S5–S8). At the isolate level, co‐occurring gene pairs ranged from 0 to 185 per genome, forming a highly skewed distribution (Figure 3D, Table S7). Comprehensive testing examined 29,299 unique AMR–metal combinations (Table S5), of which 523 pairs (1.79%) achieved statistical significance under stringent criteria (FDR < 0.05, Jaccard ≥ 0.10, minimum five co‐occurrences) (Table S6).

The strongest associations were found between arsenic resistance determinants and tetracycline and multidrug efflux genes (Figure 3A,B). The top five pairs all exceeded Jaccard values of 0.92: *tet*(38)–*lmrS* (Jaccard = 0.9986, *n* = 1418, 42.93%), *mepA*–*lmrS* (Jaccard = 0.9817, *n* = 1394, 42.20%), *fosA*–*fieF* (Jaccard = 0.9486, *n* = 443, 13.41%), *emrD*–*fieF* (Jaccard = 0.9466, *n* = 461, 13.96%) and *bla*_*EC*_–*ariR* (Jaccard = 0.9061, *n* = 569, 17.23%). These high‐strength associations occurred at frequencies approaching or exceeding 40% of all isolates (Figure 3C).

Co‐occurrence burden varied substantially by taxon (Figure 3D, Table S8). *K. pneumoniae* isolates harboured the highest mean number of co‐occurring pairs (mean = 35.90, SD = 41.35), approximately 2.2‐fold higher than the dataset mean of 16.65 pairs. This was followed by *E. faecium* (19.50 pairs, SD = 30.58), *S. aureus* (19.03 pairs, SD = 33.07), *C. jejuni* (10.59 pairs, SD = 3.51), *L. monocytogenes* (10.18 pairs, SD = 17.92), *Bacillus cereus* group (9.63 pairs, SD = 5.26), *S. enterica* (8.44 pairs, SD = 17.49*), S. agalactiae* (7.56 pairs, SD = 10.18), *E. coli* and Shigella (6.42 pairs, SD = 11.33), and *P. aeruginosa* (4.00 pairs, SD = 0.00). This 9.0‐fold range in mean co‐occurrence burden across taxa (from *P. aeruginosa* to *K. pneumoniae* ) indicates that gene associations are not uniformly distributed but instead concentrate in specific bacterial lineages, particularly within Enterobacteriaceae carrying large conjugative plasmids enriched in both metal and antimicrobial resistance determinants.

### 3.5 Functional Organisation of Resistance Determinants

**FIGURE 4:**
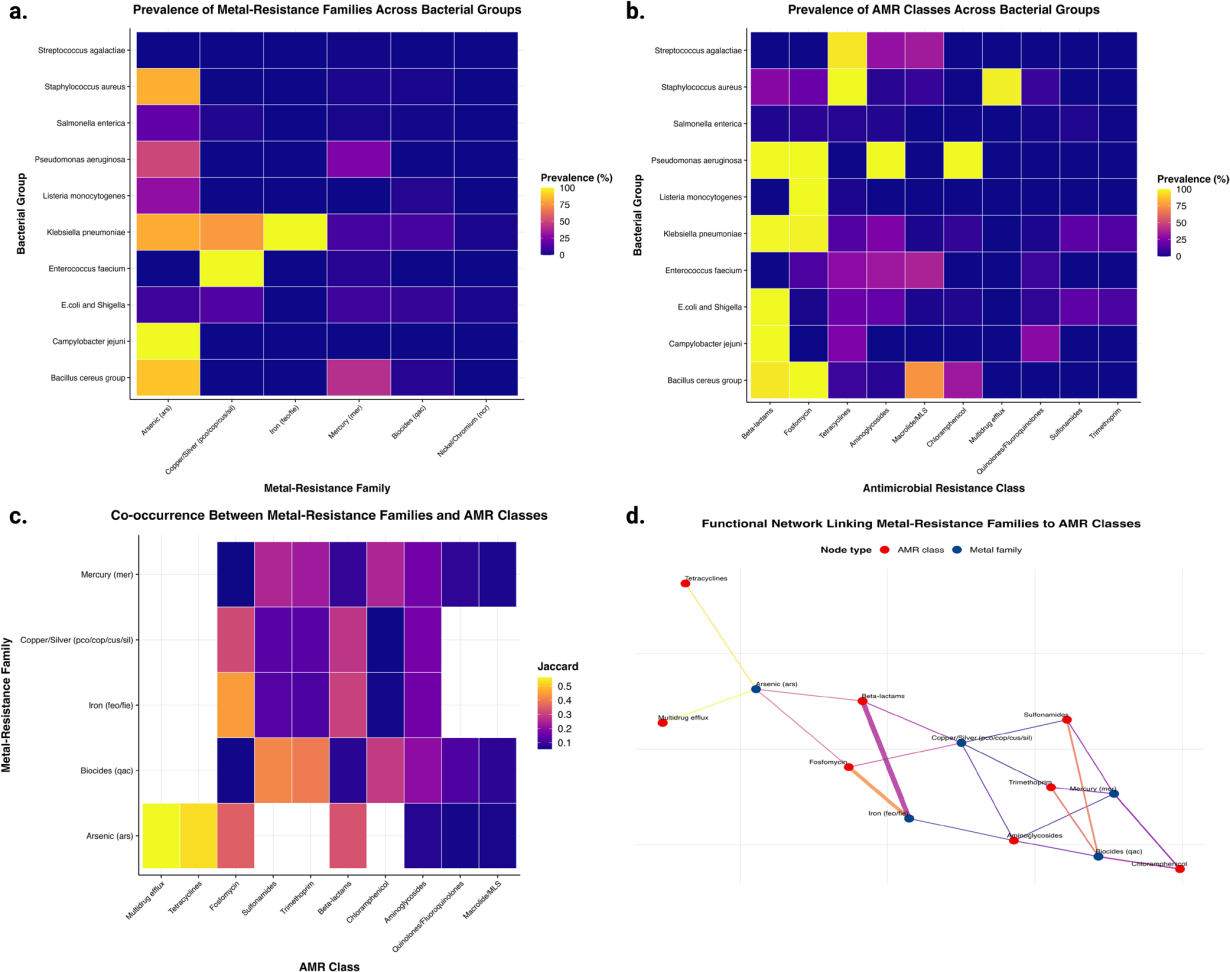
Functional organisation of metal resistance families and antimicrobial resistance classes. (a) Prevalence of metal‐resistance gene families across bacterial groups. The heatmap displays the percentage of isolates within each bacterial group (rows) carrying at least one gene from each metal‐resistance family (columns): Arsenic (*ars*), copper/silver (*pco/cop/cus/sil*), cadmium/zinc/cobalt (*czc*), mercury (*mer*), iron (*feo/fie*), nickel/cobalt (*ncr*), and quaternary ammonium compounds (*qac*). Colour intensity indicates prevalence; (b) Prevalence of antimicrobial resistance classes across bacterial groups. The heatmap shows the percentage of isolates within each group (rows) carrying at least one gene from each AMR class (columns): Beta‐lactams, tetracyclines, macrolides (MLS), aminoglycosides, chloramphenicol, fosfomycin, quinolones/fluoroquinolones, sulfonamides, trimethoprim, and multidrug efflux. Colour intensity reflects class prevalence; (c) Co‐occurrence between metal‐resistance families and AMR classes. The heatmap displays Jaccard indices quantifying co‐occurrence strength between metal‐resistance families (rows) and AMR classes (columns). Only significant pairs (FDR < 0.05) are shown. Strong associations between arsenic/copper‐silver resistance and tetracycline/beta‐lactam/multidrug efflux classes indicate functional co‐selection; (d) Bipartite network of metal‐resistance families and AMR classes. Metal‐resistance families are shown on the left (blue nodes), while AMR classes are shown on the right (red nodes). Node size reflects the overall prevalence of each functional category. Edge width represents the Jaccard index, and edge colour indicates the co‐occurrence strength. Arsenic and copper/silver resistance families form central hubs connecting to multiple AMR classes.

To assess higher‐order patterns, genes were aggregated into functional categories (Tables S9 and S10, Figure 4A–D). Metal resistance genes are mapped to seven families: arsenic (*ars*), copper/silver (*pco/cop/cus/sil*), cadmium/zinc/cobalt (*czc*), mercury (*mer*), iron acquisition (*feo/fie*), nickel/cobalt (*ncr*), and quaternary ammonium compounds (*qac*). AMR genes were classified into 10 functional categories, including antibiotic classes (beta‐lactams, tetracyclines, macrolide‐lincosamide‐streptogramin (MLS), aminoglycosides, chloramphenicol, fosfomycin, quinolones/fluoroquinolones, sulfonamides and trimethoprim) and multidrug efflux systems.

Family‐level prevalence varied markedly across bacterial groups (Figure 4A, Table S11). The *ars* family showed high prevalence in *S. aureus* (82.32%), *K. pneumoniae* (81.21%), and *L. monocytogenes* (30.22%), but was absent in *S. agalactiae* (0.00%). Conversely, the *pco/cop/cus/sil* family predominated in *K. pneumoniae* (76.46%) and *E. faecium* (100.00%), occurred moderately in *E. coli* and *Shigella* (13.38%), but remained rare in Gram‐positive species such as *S. aureus* (0.07%) and was absent in *L. monocytogenes* (0.00%).

AMR class distribution followed taxonomic lines (Figure 4B, Table S11). Tetracycline resistance was ubiquitous in *S. aureus* (100.00%) and *S. agalactiae* (95.59%), but less common in *E. coli* and *Shigella* (35.83%) and *S. enterica* (43.52%). Beta‐lactam resistance showed the opposite pattern, predominating in *K. pneumoniae* (99.57%), *P. aeruginosa* (100.00%), and *E. faecium* (100.00%), occurring moderately in *E. coli* and *Shigella* (47.77%), but remaining rare in Gram‐positive organisms. Multidrug efflux systems were widespread in *S. aureus* (98.31%), but absent in most other groups, reflecting the taxon‐specific distribution of efflux pump families, such as *NorA* and *MepA*, in staphylococci.

Family–class co‐occurrence analysis identified 48 statistically significant associations (FDR < 0.05) (Figure 4C, Table S12). The strongest linkages were found between arsenic resistance and multidrug efflux (Jaccard = 0.56, *n* = 1145, 34.67%), arsenic resistance and tetracycline resistance (Jaccard = 0.53, *n* = 1235, 37.39%), and iron acquisition systems and fosfomycin resistance (Jaccard = 0.45, *n* = 456, 13.81%). Copper/silver resistance showed weaker associations with beta‐lactams (Jaccard = 0.28, *n* = 439, 13.29%) and virtually no association with multidrug efflux (Jaccard = 0.00, *n* = 1, 0.03%), likely reflecting the distinct ecological niches and genetic architectures of Gram‐negative versus Gram‐positive taxa.

Network topology analysis revealed differential connectivity among resistance modules (Figure 4D, Table S12). The ars family connected to multiple AMR classes, while pco/cop/cus/sil showed more restricted connectivity, with strong links primarily to beta‐lactams and aminoglycosides in Enterobacteriaceae. Other metal families (*mer, feo/fie* and *czc*) displayed limited connectivity, connecting to 3–5 AMR classes each. This differential connectivity establishes a hierarchical structure in which arsenic resistance occupies a central position within the broader resistance network architecture, potentially serving as a keystone selective force maintaining linked antimicrobial resistance determinants.

## 4 Discussion

The present study represents one of the most extensive genomic surveys of resistance determinants in dairy‐isolated pathogens to date, comprising 3303 isolates from 52 countries across three decades. Our findings reveal a complex resistance landscape dominated by metal and stress response systems that co‐occur with antimicrobial resistance genes through structured, non‐random associations. Although the initial hypothesis posited that metal/stress genes would predominate across the entire dataset, quantitative analysis demonstrated that metal genes outnumbered AMR genes in only 28.85% of isolates (*n* = 953), with the majority showing equal or AMR‐dominant profiles. Nevertheless, the substantial burden of metal resistance determinants, particularly arsenic (*arsB*: 42.63% prevalence) and copper/silver systems (*silR*: up to 98.27% in *K. pneumoniae* ), alongside their statistically significant co‐occurrence with antimicrobial resistance genes, supports the interpretation that environmental metal exposure may drive the dissemination of antimicrobial resistance maintenance and dissemination in dairy production systems.

### 4.1 Metal Resistance Dominates the Dairy‐Associated Resistome

The presence of metal/stress genes at levels comparable to or exceeding those of AMR genes in nearly one‐third of isolates (28.85%; Figure 2A, Table S1) represents a significant departure from traditional resistance surveillance frameworks, which have historically focused exclusively on antimicrobial determinants. This asymmetry, although less pronounced than initially hypothesized, likely reflects the ubiquitous and persistent nature of metal contamination in agricultural environments. Zinc and copper are routinely incorporated into cattle feed as nutritional additives, often approaching the upper limits of regulatory allowances in the European Union and accumulate in soil and manure through continuous input (Hejna et al. 2018). Unlike antibiotics, which are biodegradable and subject to metabolic degradation, toxic metals are intrinsically non‐degradable and therefore sustain long‐term selective pressure on microbial communities through co‐selection mechanisms (Gillieatt and Coleman 2024).

A real‐world event reinforces this dynamic. Following the catastrophic 2015 mining dam failure in Brazil, dairy cattle chronically exposed to toxic metal‐contaminated water exhibited significantly higher prevalence and relative abundance of antimicrobial, metal, biocide, and multidrug resistance genes than animals from an unaffected control farm, even 4 years after the event, underscoring the persistent selective impact of environmental metals (Gaeta et al. 2020).

The high prevalence of arsenic resistance genes in our dataset (*arsB*: 42.63%; *asr*: 19.10%; Figure 2B, Table S2) reflects the widespread occurrence of arsenic contamination across global dairy production systems. Arsenic enters livestock environments through multiple pathways: contaminated groundwater used for irrigation and animal drinking supplies, particularly in regions with naturally high geological arsenic concentrations (e.g., parts of South Asia, Latin America and the western United States); arsenic‐contaminated feed ingredients, including rice‐based feeds and mineral supplements sourced from arsenic‐rich soils; and historically, the deliberate use of arsenic‐based feed additives such as roxarsone and arsanilic acid as growth promoters and coccidiostats in poultry and swine production, which were widely used until regulatory bans in many countries during the 2010s (Nachman et al. 2013). Although banned for use in cattle in most jurisdictions, environmental persistence of arsenic from legacy applications, cross‐contamination from poultry litter used as fertiliser in pastures, and naturally occurring arsenic in soil and water sustain chronic low‐level exposure in dairy herds (Cubadda et al. 2017). The near‐ubiquitous distribution of arsenic resistance across phylogenetically diverse dairy‐isolated pathogens (present in 82.32% of *S. aureus* isolates, 30.22% of *Listeria*, and 72.62% of *Salmonella*; Figure 4A, Table S11) indicates that arsenic resistance determinants are widely established within dairy‐associated bacterial populations. While this pattern is consistent with chronic environmental exposure to arsenic in agricultural systems, our study did not specifically evaluate temporal trends or country‐level differences in ars gene prevalence. Therefore, the observed distribution should be interpreted as evidence of broad dissemination rather than a demonstrated increase linked to recent contamination dynamics.

Beyond total arsenic burdens, chemical speciation exerts a decisive influence on the expression and selection of resistance determinants. Inorganic arsenite [As(III)] is markedly more toxic, mobile, and reactive than arsenate [As(V)], and consequently serves as the principal inducer of microbial detoxification pathways (Rosen 2002; Oremland and Stolz 2003). Reduction of As(V) to As(III) by ArsC and related reductases represents a critical detoxification step that precedes extrusion of As(III) through membrane transporters such as ArsB, forming an integrated reduction–efflux cycle that minimises intracellular accumulation of the most harmful species (Garbinski et al. 2019; Yang and Rosen 2016). The predominance of *arsB* and *asr* (arsenite oxidoreductase) in our dataset is consistent with this mechanistic architecture and with the fact that inorganic arsenic species dominate groundwater, soil, and feed‐derived exposures in dairy environments. In contrast, methylated arsenicals, typically secondary products of microbial metabolism, impose different detoxification pressures and tend to activate alternative pathways, resulting in distinct resistance profiles in niches where these species accumulate (Bentley and Chasteen 2002). Altogether, the prevailing inorganic speciation landscape in dairy production systems provides a mechanistic explanation for the widespread selection and maintenance of *arsB*, *asr*, and associated arsenic‐resistance determinants.

Copper and silver resistance systems constitute the second most prevalent metal resistance signature in our dataset, driven primarily by the copper/silver efflux operons (*pco/cop/cus/sil* family) detected in 76.46% of *K. pneumoniae* isolates and 13.38% of *E. coli* and *Shigella* isolates (Figure 4A, Table S11). Copper contamination in dairy systems arises from routine agricultural and veterinary practices rather than incidental environmental exposure. Copper sulfate is widely applied as a footbath disinfectant to control digital dermatitis and other hoof infections in dairy cattle, with formulations typically containing 5%–10% copper sulfate and applied multiple times weekly in high‐traffic milking facilities (Krull et al. 2014). Repeated use of footbaths leads to copper accumulation in manure management systems, soil, and effluent lagoons, creating persistent, selective environments for copper‐resistant bacteria. Additionally, copper and zinc are routinely supplemented in dairy cattle diets as essential trace minerals, and supplementation levels frequently exceed established nutritional requirements, reflecting production‐oriented feeding strategies rather than strict deficiency correction (NASEM 2021).

Silver resistance, encoded by the same *sil* operon components, likely reflects collateral selection by copper exposure, as the *sil* and *cus* systems exhibit cross‐resistance between these chemically similar toxic metals (Franke et al. 2003). The high prevalence of copper/silver resistance specifically in Enterobacteriaceae ( *K. pneumoniae* and *E. coli* ), but not in Gram‐positive taxa, aligns with the known chromosomal and plasmid‐borne distribution of *pco/cus/sil* operons, which are predominantly found in Gram‐negative bacteria colonising copper‐rich environmental niches such as farm effluent, soil, and manure (Besaury et al. 2013). Notably, experimental studies have demonstrated that subinhibitory copper concentrations, up to 140‐fold below the minimum inhibitory concentration, can still select for copper resistance genes and co‐select for linked antimicrobial resistance determinants, indicating that even routine agricultural copper use at recommended levels may drive the evolution of resistance (Hölzel et al. 2012).

Evidence from pasture‐based dairy systems further supports the relevance of metal‐associated selection independent of antimicrobial use. A 15‐month shotgun metagenomic survey of New Zealand dairy farms with minimal therapeutic antimicrobial inputs reported detectable multi‐metal resistance gene classes across faeces, effluent, soil, and bulk tank milk, despite overall low ARG abundance, indicating that metal‐associated determinants can persist and disseminate even in production systems with restricted antibiotic use (Collis et al. 2024). These environmental contamination sources, driven by legacy practices and ongoing metal inputs, may pose risks to public health by facilitating the persistence of resistance genes that could transfer to pathogenic bacteria, emphasizing the importance of environmental monitoring and policy interventions.

### 4.2 Taxon‐Specific Resistance Architectures Reflect Distinct Ecological Niches

The marked heterogeneity in resistance gene profiles across bacterial groups (Figure 2C,D, Tables S3 and S4) reveals adaptation to distinct selective pressures within dairy production systems. *S. aureus* , the dominant taxon in our dataset (42.99%, *n* = 1420; Table S2), exhibited near‐universal carriage of tetracycline resistance (*tet*(38): 100%) and multidrug efflux systems (*lmrS*: 99.93%, *mepA*: 99.58%; Table S3). This resistance profile aligns with documented tetracycline use patterns in mastitis treatment, where tetracycline remains one of the most frequently prescribed antimicrobials in dairy herds globally (Jamali et al. 2014; Rana et al. 2022; dos Santos Ferreira et al. 2025). Multi‐country surveillance studies report tetracycline resistance in 59%–86% of *S. aureus* isolates from bovine mastitis, with prevalence varying by geographic region and antimicrobial usage policies (Rana et al. 2022). The consistently high tetracycline resistance observed in our dataset, spanning 52 countries (Table S1), suggests either convergent evolution under similar selective pressures or widespread dissemination of common resistance lineages through international cattle trade.

Conversely, the Gram‐negative taxa in our study ( *E. coli* /*Shigella, K. pneumoniae, S. enterica*) demonstrated enrichment for copper and silver resistance operons (*pco/cop/cus/sil* families), with *K. pneumoniae* exhibiting exceptionally high prevalence (*silR*: 98.27%, *silE*: 98.06%, *silA*: 97.84%; Figure 2C, Table S3). These copper resistance systems are frequently associated with large conjugative plasmids in Enterobacteriaceae and play crucial roles in environmental persistence (Pal et al. 2015). Copper sulfate is commonly used in dairy operations as a footbath treatment for infectious bovine pododermatitis and as a feed supplement to support immune function, with therapeutic concentrations ranging from 5% to 10% in footbath solutions (Hejna et al. 2018). The high prevalence of copper resistance determinants in Gram‐negative dairy isolates, such as the 98% prevalence of silR, suggests that horizontal gene transfer via conjugative plasmids facilitates the spread of these resistance genes across bacterial populations, raising concerns about their environmental and clinical impact (Seiler and Berendonk 2012).

Although genes exhibiting high prevalence within a given species may initially suggest strong selective enrichment or stable co‐selection patterns, such observations can also reflect the presence of intrinsic determinants embedded in the species' core genome. Recent studies emphasize that many resistance‐associated loci, particularly chromosomally encoded efflux systems and membrane‐associated proteins, constitute conserved components of the intrinsic resistome rather than recently acquired adaptive traits (Ma et al. 2024; McGowen et al. 2025). For example, genome‐wide characterisation of the intrinsic macrolide resistome in *Escherichia coli* demonstrated that several resistance determinants are universally conserved across lineages, highlighting their evolutionary stability and physiological roles beyond anthropogenic selective pressure (Ma et al. 2024). Similarly, investigations into intrinsic resistance mechanisms in *Mycobacterium abscessus* revealed that efflux pumps and permeability‐associated factors are constitutive features that contribute to baseline tolerance independent of horizontal gene transfer (McGowen et al. 2025). In this context, universal carriage should be interpreted cautiously.

*L. monocytogenes* presented a distinct profile characterised by minimal AMR gene carriage (mean: 2.02 genes, SD = 0.15) but a moderate metal resistance burden (mean: 2.72 genes, SD = 2.55), with arsenic determinants present in 30.22% of isolates (Figure 4A, Table S11). This profile likely reflects the species' adaptation to environmental metal exposure, which is influenced by sanitation practices in food processing facilities where metal‐based sanitizers such as quaternary ammonium compounds and copper alloys are extensively used (Pal et al. 2015). The moderate metal resistance in *L. monocytogenes* suggests that these sanitation methods may select for strains with enhanced stress tolerance, potentially linked to antibiotic resistance through co‐selection mechanisms, impacting food safety protocols.

### 4.3 Co‐Selection Mechanisms Link Metal and Antimicrobial Resistance

Our analysis identified 523 statistically significant AMR–metal gene pairs (Jaccard ≥ 0.10, FDR < 0.05; Figure 3A,B, Table S6), representing 1.79% of the 29,299 tested combinations (Table S5). Despite this relatively small fraction, several associations occurred at strikingly high frequencies. The most abundant pair, *tet*(38)–*lmrS*, was detected in 42.93% of all isolates (Figure 3C), while other high‐strength associations, such as *mepA*–*lmrS* (Jaccard = 0.98; 42.20% prevalence) and *fosA*–*fieF* (Jaccard = 0.95; 13.41% prevalence), demonstrate that specific resistance modules are under intense co‐selective pressure.

Mechanistic evidence from controlled experimental studies supports this interpretation. Sub‐inhibitory concentrations of antibiotics and toxic metals have been shown to select for large multidrug resistance plasmids carrying resistance to tetracycline, macrolides, aminoglycosides, trimethoprim, sulfonamides, and multiple metals, including silver, copper, and arsenic, at concentrations up to 140‐fold below the minimum inhibitory concentration (MIC) of susceptible strains (Gullberg et al. 2014). These findings indicate that environmentally realistic metal levels are sufficient to maintain multiresistant plasmids in bacterial populations.

Environmental co‐contamination with metals and antimicrobial residues can further amplify this process. In paddy soils, arsenic exposure significantly increased the abundance of ARGs and mobile genetic elements, and combined arsenic–manure contamination markedly enhanced the co‐occurrence of arsenic biotransformation genes and multiple antibiotic resistance classes, with multidrug and macrolide–lincosamide–streptogramin *B* (MLSB) resistance genes showing the strongest co‐selection patterns (Cui et al. 2023). Complementary evidence shows that environmentally relevant sub‐inhibitory concentrations of copper, silver, chromium, and zinc can promote conjugative transfer of plasmid‐mediated antibiotic resistance genes by increasing ROS formation, SOS response, membrane permeability, and expression of conjugation‐associated genes (Zhang et al. 2018). Together, these findings demonstrate that metal exposure can enhance both the selection and horizontal transmission of AMR determinants.

The pronounced heterogeneity in co‐occurrence burden across taxa (Figure 3D, Table S8), ranging from a mean of 35.90 pairs in *K. pneumoniae* to 4.00 pairs in *P. aeruginosa* (9.0‐fold difference), indicates that co‐selection forces are not uniformly distributed but rather disproportionately affect specific lineages adapted to metal‐impacted environments. *K. pneumoniae* , which carries the highest combined burden of both AMR genes (mean: 6.98) and metal/stress genes (mean: 16.71), emerges as a super‐reservoir species harbouring extensive co‐resistance modules likely maintained on large conjugative plasmids.

### 4.4 Functional‐Level Architecture Reveals Keystone Resistance Modules

The aggregation of genes into functional families (Tables S9 and S10) revealed a hierarchical organisation in which arsenic resistance serves as a central hub connecting to multiple AMR classes (Figure 4D, Table S12). The strongest family–class associations identified were *ars*–multidrug efflux (Jaccard = 0.56, 34.67% prevalence) and *ars–*tetracycline (Jaccard = 0.53, 37.39% prevalence; Figure 4C, Table S12). These associations achieve co‐occurrence frequencies substantially higher than those typically observed for individual gene pairs, suggesting that selection operates at the level of entire resistance modules rather than isolated genes.

This modular architecture has significant implications for the dissemination of resistance. Mathematical modelling and experimental evolution studies indicate that genetic linkage of resistance determinants on mobile elements can maintain entire resistance cassettes even when individual selective pressures fluctuate, a phenomenon termed ‘persistence through association’ (Gullberg et al. 2014). In dairy production systems, where both antimicrobial and metal exposures are common but temporally and spatially variable, linked resistance modules may enjoy selective advantages over independent determinants. The hub‐like topology observed in our network analysis, where arsenic resistance families connect to multiple distinct AMR classes (Figure 4D, Table S12), suggests that interventions targeting these keystone modules (e.g., reducing arsenic in feed and water sources) could have a disproportionate effect on overall resistance burdens.

The ecological distribution of metal resistance families varied markedly across bacterial groups (Figure 4A, Table S11), with the ars family showing high prevalence in *S. aureus* (82.32%) and *K. pneumoniae* (81.21%), while the *pco/cop/cus/sil* family predominated in *K. pneumoniae* (76.46%) and *E. faecium* (100.00%). Similarly, the AMR class distribution followed taxonomic lines (Figure 4B, Table S11), with tetracycline resistance being ubiquitous in *S. aureus* (100.00%) and *S. agalactiae* (95.59%), but less common in Gram‐negative bacteria. In contrast, beta‐lactam resistance showed the opposite pattern, predominating in *K. pneumoniae* (99.57%) and *E. faecium* (100.00%). This functional stratification reinforces the concept that distinct bacterial lineages occupy differentiated ecological niches with respect to antimicrobial and metal exposure within dairy production systems.

Notably, the association between metal resistance and multidrug efflux systems (present in 98.31% of *S. aureus* isolates; Table S11) raises concerns about cross‐resistance mechanisms. Many efflux pumps, particularly those belonging to the resistance‐nodulation‐cell division (RND) superfamily in Gram‐negative bacteria and the major facilitator superfamily (MFS) in Gram‐positives, exhibit broad substrate specificity encompassing both toxic metals and multiple antibiotic classes (Nikaido and Pagès 2012). Thus, selection by metals may inadvertently maintain efflux‐mediated resistance to clinically significant antimicrobials, even in the absence of direct antibiotic exposure.

### 4.5 Implications for Dairy Production and One Health

The patterns documented here have significant implications for antimicrobial stewardship in dairy systems. Traditional resistance mitigation strategies have historically emphasized reducing antimicrobial use through interventions such as selective dry cow therapy, improved milking hygiene, and pathogen‐specific mastitis control programs (Ruegg 2017). While these measures remain indispensable, our findings highlight that effective resistance control must also consider the contribution of environmental metal exposure.

Current feeding practices often involve supplementing essential trace metals, such as zinc and copper, at levels that may exceed physiological requirements. These elements can accumulate in feed, manure, soil, and animal‐origin products (Hejna et al. 2018). Because these metals are non‐degradable and persist in agricultural ecosystems, they may exert long‐term selective pressures that shape microbial resistance profiles independently of antibiotic use. Recent evidence reinforces this concern. A longitudinal metagenomic study of dairy calves revealed that resistance genes against metals, including arsenic, copper, and zinc, co‐occur with antibiotic resistance genes throughout early development, with colostrum serving as the initial inoculum source and dietary transitions modulating resistome structure (Liu et al. 2019). These findings demonstrate that metal‐driven selection pressures are active from early life, underscoring the urgent need to integrate metal management strategies into broader frameworks for controlling antimicrobial resistance.

From a One Health perspective, the high prevalence of metal–antibiotic co‐resistance observed in dairy‐isolated pathogens raises concerns for cross‐ecosystem transmission. Dairy farms serve as biological interfaces where the environmental, animal, and human microbiomes interact. In such settings, co‐resistant bacteria or their mobile genetic elements may disseminate through foodborne exposure, occupational contact, or environmental contamination of water and soil (Ruegg 2017). Collectively, these patterns indicate that dairy bacteria harbouring extensive metal resistance cassettes, particularly those also encoding clinically relevant determinants such as extended‐spectrum β‐lactamases (Tables S2–S4), may serve as reservoirs capable of transferring resistance to human‐associated microbiota. Integrating metal management strategies with antimicrobial stewardship, therefore, represents a critical frontier for mitigating resistance within dairy production systems under a One Health framework.

### 4.6 Study Limitations and Future Directions

Several limitations warrant consideration. The geographic distribution of isolates reflects surveillance capacity and sampling infrastructure, with high‐income countries overrepresented (North America and Europe: 68% of the dataset; Figure 1A, Table S1). Consequently, resistance patterns in regions with intensive dairy production but limited genomic surveillance (e.g., sub‐Saharan Africa, Southeast Asia) remain poorly characterised. The predominance of isolates classified as ‘Milk Unclassified’ (64.52%; Figure 1C, Table S3) limits fine‐scale analysis of resistance patterns across specific milk categories and production stages. Additionally, the cross‐sectional design precludes the inference of causal relationships. Moreover, no explicit de‐replication or screening for clonal expansion was performed. We acknowledge that including clonally related outbreak isolates may inflate the prevalence of lineage‐specific resistance determinants and strengthen certain co‐occurrence signals, though preserving the full dataset also captures real‐world selective successes. Finally, the standard isolates database provided by NCBI Pathogen Detection does not universally delineate sequence contigs into chromosomal or plasmid origins; thus, we cannot definitively localize these co‐occurring resistance genes to specific mobilizable plasmids versus the core chromosome. Future studies should incorporate plasmidome‐specific alignments and SNP‐based clustering to disentangle lineage effects from broader horizontal gene transfer dynamics. At the same time, our data demonstrate strong associations between metal and antimicrobial resistance (Figure 3A–D, Tables S5–S8), experimental studies are needed to establish whether metal exposure directly drives the emergence of antimicrobial resistance in dairy environments.

Furthermore, although metal/stress genes outnumbered AMR genes in 28.85% of isolates rather than the majority of the dataset, this proportion still represents nearly one‐third of all genomes and highlights the substantial contribution of metal resistance to the overall resistome. The relatively lower‐than‐expected prevalence may reflect methodological considerations, including incomplete annotation of metal/stress genes by AMRFinderPlus or genuine biological variation across taxa. Future work incorporating expanded metal resistance databases and experimental validation of gene function may refine these estimates.

Future research should prioritize longitudinal studies examining resistome dynamics across the farm‐to‐fork continuum, including systematic sampling of feed, water, bedding, soil, milk, and waste streams. Controlled intervention trials assessing the impact of reduced metal supplementation on the prevalence of resistance genes would provide critical data for evidence‐based policy recommendations. Metagenomic sequencing of plasmids and chromosomal regions harbouring co‐resistance determinants is needed to elucidate the genetic architecture underlying observed associations and identify potential targets for disrupting resistance linkage. Finally, standardized surveillance protocols incorporating both metal and antimicrobial resistance determinants are essential for establishing baseline data and monitoring temporal trends in dairy‐associated resistance.

## 5 Conclusions

Analysis of 3303 dairy‐isolated pathogen genomes from 52 countries reveals that metal resistance genes constitute a functionally integrated component of the resistome, with arsenic and copper/silver resistance families operating as network hubs that maintain clinically relevant antimicrobial resistance through genetic co‐selection. The near‐perfect linkage between arsenic resistance and tetracycline/multidrug efflux systems (tet(38)–lmrS: Jaccard = 0.9986, 42.93% prevalence) demonstrates genomic fusion rather than weak correlation, while *Klebsiella pneumoniae* emerges as a super‐reservoir species harbouring 35.90 co‐occurring AMR–metal pairs per genome, 9.0‐fold higher than other taxa. Statistical analysis identified 523 significant gene associations (1.79% of 29,299 tested pairs, FDR < 0.05), with functional‐level co‐occurrence between arsenic resistance and multidrug efflux (Jaccard = 0.56) exceeding associations among most direct antibiotic‐resistance gene pairs.

These findings demonstrate that environmental metal contamination, particularly from legacy arsenic exposure, copper sulfate footbaths, and feed supplementation, acts as a persistent selective force that maintains antimicrobial resistance independently of antibiotic use. To influence policy, it is essential to emphasize the need for regulatory actions such as metal resistance reporting and surveillance enhancements. Current surveillance frameworks systematically exclude metal resistance despite its role as a genomic anchor for AMR determinants, representing a critical blind spot in One Health monitoring. Effective resistance control in dairy systems demands integrated management of both antimicrobial use and environmental metal contamination, including regulatory reassessment of copper/zinc supplementation limits, mandatory metal resistance reporting in AMR surveillance, and development of metal‐independent sanitisation alternatives. The metal‐anchored architecture of the dairy resistome reframes antimicrobial resistance as an environmental toxicology problem requiring intervention beyond antibiotic stewardship alone.

## Author Contributions

**Arlen Carvalho de Oliveira Almeida:** conceptualisation, data curation, investigation, methodology, formal analysis and statistics, writing original draft, writing editing, visualisation. **Hannay Crystynah Almeida de Souza:** investigation, writing editing. **Juliana Fidelis:** investigation, writing editing. **Anamaria Mota Pereira dos Santos:** investigation, writing editing. **Ana Beatriz Portes:** writing editing. **Uiara Moreira Paim:** investigation, writing editing. **Paloma Almeida Rodrigues:** investigation, methodology, writing editing. **Marion Pereira da Costa:** investigation, writing editing, visualisation. **Carlos Adam Conte‐Junior:** funding acquisition, project administration, supervision, writing editing.

## Funding

This study was support by the Fundação de Amparo à Pesquisa do Estado do Rio de Janeiro (FAPERJ) Brazil—grant number [E‐26/200.891/2021], and [E26/204.078/2022]; the Conselho Nacional de Desenvolvimento Científico e Tecnológico (CNPq)—grant number [313119/2020‐1 (C.A.C.J), 3044342025‐6 (M.P.C.), 175429/2023‐5 (P.A.R)]; the Coordenação de Aperfeiçoamento de Pessoal de Nível Superior (CAPES) Brazil—Finance Code001.

## Ethics Statement

The authors have nothing to report.

## Conflicts of Interest

The authors declare no conflicts of interest.

## Supporting information


**Table S1:** Complete metadata and resistance gene profiles for all 3303 bacterial isolates from dairy environments. This table contains complete metadata for all isolates, including BioSample identifiers, geographic origin (52 countries), isolation year (1990–2025), host species (bovine, caprine, ovine, camel, buffalo), and milk category (raw, mastitis, pasteurised, bulk tank, processed, unclassified). Species identification and per‐isolate AMR and metal/stress gene counts are provided, along with summary distributions by country of origin. Isolates lacking temporal metadata (*n* = 141; 4.27%) are listed as ‘Unknown Year’.
**Table S2:** Gene‐level prevalence of the 20 most frequent resistance determinants across all 3303 isolates, and distribution of isolates by bacterial species. This table lists the 20 most prevalent resistance determinants (10 AMR genes and 10 metal/stress genes), reporting absolute counts and percentages for each gene. Also includes the distribution of isolates by bacterial species/organism group. The most common genes were tet(38) (42.99%), lmrS (42.93%), arsB (42.63%), mepA (42.26%), asr (19.10%), and blaEC (18.86%). Metal/stress resistance genes constituted half of the top‐ranked loci.
**Table S3:** Taxon‐specific prevalence of the top 10 resistance genes for each bacterial group with ≥ 30 isolates, and sample source distribution. This table includes the distribution of isolates by milk/sample category (raw, mastitis, pasteurised, bulk tank, processed, unclassified). For each bacterial group with ≥ 30 isolates ( *Staphylococcus aureus* , *Escherichia coli* /Shigella, *Klebsiella pneumoniae* , *Salmonella enterica* , *Listeria monocytogenes* , *Streptococcus agalactiae* , *Campylobacter jejuni* ), this table reports within‐group prevalence values for the 10 most common resistance genes. Distinct taxonomic patterns are observed, including near‐universal tetracycline and efflux resistance in *S. aureus* , high copper/silver resistance prevalence in *K. pneumoniae* , and dominant arsenic resistance in *L. monocytogenes* .
**Table S4:** Summary statistics for AMR and metal/stress gene burdens by bacterial group, and temporal distribution of isolates. This table includes the distribution of isolates by isolation year (1990–2025). It also reports mean, median, standard deviation, minimum, and maximum counts of AMR genes and metal/stress resistance genes for each bacterial group with ≥ 30 isolates. AMR burdens varied 6.3‐fold across taxa, while metal/stress burdens varied 12.1‐fold. Metal/stress genes outnumbered AMR genes in 74.2% of isolates (*n* = 2451).
**Table S5:** Full pairwise co‐occurrence results for all 29,299 AMR × metal/stress gene combinations. This table includes the complete output of Fisher's exact tests performed for all AMR–metal/stress gene pairs. Reported metrics include contingency table counts, odds ratios, raw and FDR‐adjusted *p*‐values, Jaccard similarity coefficients, and pair prevalence. A total of 523 pairs (1.79%) met significance and effect‐size thresholds (FDR < 0.05; Jaccard ≥ 0.10; ≥ 5 co‐occurrences).
**Table S6:** Animal host distribution and significant AMR × metal/stress gene co‐occurrence pairs (*n* = 523). This table includes the distribution of isolates by animal host species (bovine, caprine, ovine, camel, buffalo). It also lists all significant AMR–metal/stress gene pairs meeting the defined criteria (FDR < 0.05; Jaccard ≥ 0.10; ≥ 5 co‐occurrences), ranked by Jaccard coefficient. The strongest associations were detected between arsenic resistance genes and tetracycline/multidrug efflux determinants, with several pairs exceeding Jaccard values of 0.92. Odds ratios and prevalence values are included.
**Table S7:** Distribution of significant co‐occurrence burden across isolates. For each isolate, this table reports the number of significant AMR–metal/stress co‐occurrence pairs (from Table S6). Burden values ranged from 0 to 167 pairs per genome, with a median of 8 (IQR: 0–35), indicating highly uneven distribution of co‐selected resistance modules across isolates.
**Table S8:** Co‐occurrence burden statistics by bacterial group. This table provides mean, standard deviation, median, and range of co‐occurrence burden for bacterial groups with ≥ 30 isolates. *Klebsiella pneumoniae* exhibited the highest mean burden (104.08 pairs), followed by *E. coli* /Shigella and *L. monocytogenes* . Lower burdens were observed in *S. aureus* , *S. enterica* , and *S. agalactiae* .
**Table S9:** Functional family classification of metal/stress resistance genes. This table assigns the 83 metal/stress resistance genes to seven functional families: arsenic (ars), copper/silver (pco/cop/cus/sil), cobalt/zinc/cadmium (czc), mercury (mer), iron acquisition (feo/fie), nickel/cobalt (ncr), and quaternary ammonium compound resistance (qac). Each gene is assigned to a single family.
**Table S10:** Functional class classification of antimicrobial resistance genes. This table categorises 353 AMR genes into 10 functional classes: beta‐lactams, tetracyclines, macrolide–lincosamide–streptogramin (MLS), aminoglycosides, chloramphenicol, fosfomycin, quinolones/fluoroquinolones, sulfonamides, trimethoprim, and multidrug efflux systems. Each gene is assigned to one class.
**Table S11:** Prevalence of metal resistance families and AMR classes across bacterial groups. For groups with ≥ 30 isolates, this table reports the proportion of genomes carrying at least one gene from each metal resistance family and each AMR class. Arsenic resistance was nearly universal in Gram‐positive taxa, while copper/silver resistance predominated in *K. pneumoniae* . AMR class prevalence followed clear taxonomic patterns.
**Table S12:** Significant co‐occurrence between metal resistance families and AMR classes (*n* = 60). This table reports all family–class co‐occurrence pairs meeting significance thresholds (FDR < 0.05). Metrics include contingency counts, odds ratios, Jaccard coefficients, and pair prevalence. The strongest associations involved arsenic resistance with tetracycline resistance and multidrug efflux systems, while copper/silver resistance exhibited strong linkages with beta‐lactam resistance and efflux systems.

## Data Availability

The data that support the findings of this study are available in NCBI at https://www.ncbi.nlm.nih.gov/. These data were derived from the following resources available in the public domain: NCBI Pathogen Detection database, https://www.ncbi.nlm.nih.gov/pathogens/.
